# Probing the Intracellular Bio-Nano Interface in Different Cell Lines with Gold Nanostars

**DOI:** 10.3390/nano11051183

**Published:** 2021-04-30

**Authors:** Cecilia Spedalieri, Gergo Péter Szekeres, Stephan Werner, Peter Guttmann, Janina Kneipp

**Affiliations:** 1 Department of Chemistry, Humboldt-Universität zu Berlin, Brook-Taylor-Str. 2, 12489 Berlin, Germany; cecilia.spedalieri@hu-berlin.de (C.S.); gpszekeres@fhi-berlin.mpg.de (G.P.S.); 2 School of Analytical Sciences Adlershof, Humboldt-Universität zu Berlin, Albert-Einstein-Str. 5-9, 12489 Berlin, Germany; 3 Department X-ray Microscopy, Helmholtz-Zentrum Berlin für Materialien und Energie GmbH, Albert-Einstein-Str. 15, 12489 Berlin, Germany; stephan.werner@helmholtz-berlin.de (S.W.); peter.guttmann@helmholtz-berlin.de (P.G.)

**Keywords:** gold nanostars, SERS, nanoparticle uptake, nanotomography, endocytosis, protein corona, HCT-116, J774, 3T3

## Abstract

Gold nanostars are a versatile plasmonic nanomaterial with many applications in bioanalysis. Their interactions with animal cells of three different cell lines are studied here at the molecular and ultrastructural level at an early stage of endolysosomal processing. Using the gold nanostars themselves as substrate for surface-enhanced Raman scattering, their protein corona and the molecules in the endolysosomal environment were characterized. Localization, morphology, and size of the nanostar aggregates in the endolysosomal compartment of the cells were probed by cryo soft-X-ray nanotomography. The processing of the nanostars by macrophages of cell line J774 differed greatly from that in the fibroblast cell line 3T3 and in the epithelial cell line HCT-116, and the structure and composition of the biomolecular corona was found to resemble that of spherical gold nanoparticles in the same cells. Data obtained with gold nanostars of varied morphology indicate that the biomolecular interactions at the surface in vivo are influenced by the spike length, with increased interaction with hydrophobic groups of proteins and lipids for longer spike lengths, and independent of the cell line. The results will support optimized nanostar synthesis and delivery for sensing, imaging, and theranostics.

## 1. Introduction

Gold nanostars have unique properties that render them extremely suitable for biophotonic and theranostic applications. The plasmon resonances of these anisotropic nanostructures can be tuned in the visible and near-infrared wavelength range by modifying their size and spike morphology [1,2]. Their optical properties are specifically suited for imaging [3,4], biosensing [5,6,7], and photothermal applications [8,9,10]. The unique geometry of plasmonic nanostars enables versatile field enhancement [11,12], that allows for efficient metal-enhanced fluorescence [13] and a high electromagnetic contribution to surface-enhanced Raman scattering (SERS) [1,14]. The latter enables their application not only as SERS tags with reporters for imaging [15,16] and both extracellular [17,18] and intracellular [19,20] sensing, but also as SERS probes of biomolecules [21] and of the intrinsic biochemistry in living cells [22,23].

Apart from size [24], shape [25,26,27], and surface chemistry [25,28] of the pristine nanostructures, their protein corona has an impact on the uptake and processing by animal cells, and therefore on potential applications [29,30]. In the case of plasmonic nanoparticles, SERS has been shown as a very useful tool to probe the biomolecular corona and to characterize it in great detail even inside living cells [31,32]. Specifically, a better understanding is being gained on the surface composition of gold nanostructures in the course of the endolysosomal pathway when they are delivered into the cells through the culture medium [32,33,34,35,36]. We have recently shown that the biomolecular surface composition of gold nanostars can be characterized in living cells, that it varies depending on the morphological properties of the nanostars, and that it leads to different intracellular processing of the nanostars [23]. Nevertheless, data in this work [23] were obtained from cell lines that are known to preferentially take up nanoparticles of this size range by clathrin-mediated or calveolar-mediated endocytosis [37].

It is known that the intracellular processing of nanomaterials is also strongly determined by the type of cell [31,33,38]. In more complex biological systems, other cell types, such as macrophages, serving as the first ‘line of defense’ in vivo, may determine the applicability of the nanostructures, and therefore understanding their response is of great interest in nanotechnological applications [39,40]. Macrophage cells can enable additional nanoparticle uptake mechanisms, specifically phagocytosis [27,38,41]. Although the interaction of spherical gold nanoparticles with components in the endolysosomal compartment of macrophages has been addressed [32,35], little is known about the characteristics of these interactions with nanostructures of anisotropic morphologies, such as nanostars.

Here, we present the chemical and structural information that can be obtained from the bio–nano interface of gold nanostars in the endolysosomal system of different cell lines, specifically macrophages. Using data from SERS experiments, we will discuss differences in the interactions at the nanostars’ surface with biomolecules, and assess the intracellular localization and distribution by cryo soft X-ray nanotomography (cryo-SXT). The combination of both types of information provides a broader picture of how the different cells process the nanostars. Differently from our previous work on fibroblast and epithelial carcinoma cells, where we have discussed the interaction of gold nanostars with the cells for long incubation times [23], here we will shed light on the influence of the composition of the biomolecular corona and the nanoparticle morphology, mainly spike length, for shorter incubation times that exclude late stages of endolysosomal maturation. As the data reported here demonstrate, nanostars taken up by 3T3 fibroblast cells, HCT-116 epithelial carcinoma cells, and J774 macrophages have a protein corona of different chemical and structural properties that are a consequence of the specific endolysosomal processing of each individual cell type. Experiments with nanostars of different spike length reveal similar variations of the biomolecular surface of the nanostructures in the different cell lines, further underlining the importance of the protein corona for nanostar processing. As will be discussed for the spectral differences in the cell lines, the cell line-specific processing is found for very different stages in the endolysosomal processing pathway.

## 2. Materials and Methods

### 2.1. Gold Nanostar Synthesis

Gold(III) chloride trihydrate and 4-(2-hydroxyethyl)-1-piperazineethanesulfonic acid (HEPES) were purchased from Sigma-Aldrich (Steinheim, Germany). Gold nanostars were synthesized using a published protocol [42] with modifications as described previously [23]. Briefly, HEPES buffer (pH 7.4) at a concentration of 0.375 M was prepared with Milli-Q water and filtered through a 0.22 µm-pore filter. To obtain three different final HEPES concentrations (25, 50, and 75 mM) the buffer solution was diluted with Milli-Q water up to a volume of 12 mL in glass vials. After placing them in a thermostatic bath at 22–24 °C, an aliquot of 336 µL of a 2 mM gold chloride solution was added to each vial, the solutions quickly mixed and left undisturbed in the thermostatic bath for 80 min. Then, 864 µL of the same gold solution was added to each vial, the solution quickly mixed and left undisturbed in the thermostatic bath overnight. The obtained nanostars were stored as prepared at 4 °C. Before each experiment, if a precipitate was observed, the solutions were briefly sonicated in an ultrasound bath at room temperature. For clarity, the nanostars obtained with different HEPES concentration will be abbreviated as follows: NS25 for gold nanostars synthesized with 25 mM HEPES, NS50 for gold nanostars obtained with 50 mM HEPES, and NS75 for gold nanostars prepared with 75 mM HEPES.

UV-vis-NIR spectra of the nanostars were obtained using a double-beam spectrophotometer (Jasco, Pfungstadt, Germany) in the wavelength range of 250 to 1200 nm. Quartz cuvettes of 10 mm path length were used. Transmission electron micrographs were obtained with a Tecnai G2 20 TWIN instrument operating at 200 kV acceleration voltage. Image analysis to determine particle dimensions was performed with ImageJ software [43].

### 2.2. Cell Cultures and Incubation with Nanostars

Human colorectal carcinoma cell line HCT-116 (LGC Standards, Wesel, Germany), Swiss albino mouse macrophage cell line J774, and fibroblast cell line 3T3 (both from DSMZ, Braunschweig, Germany) were cultured in Dulbecco’s Modified Eagle Medium (DMEM; Biochrom, Berlin, Germany) supplemented with 10% fetal calf serum (FCS; Biochrom, Berlin, Germany) in a humidified environment at 37 °C with 5% CO_2_.

For SERS experiments, cells were grown on glass cover slips for 24 h prior to nanostar incubation. Then, a dilution of gold nanostars in DMEM-FCS of 1:10 (nanostar concentration ~5 × 10^−12^ M) was added and cells were incubated with the nanostars for different times. Prior to SERS measurements, the cells adhering to the glass cover slips were rinsed two times with phosphate-buffered saline (PBS, Biochrom, Berlin, Germany) to eliminate remaining culture medium and nanostars, and kept in PBS. For cryo soft X-ray nanotomography, cells were grown on Formvar-coated gold grids and incubated in a similar fashion as described for SERS experiments. After the stipulated incubation time, each grid was rinsed three times with PBS, the excess of buffer was removed with a filter paper, and the grids were plunge-frozen in liquid ethane.

### 2.3. SERS Experiments

SERS spectra were measured using a single-stage spectrograph equipped with a CCD detector (Horiba, Munich, Germany) and a diode operating at 785 nm (Toptica, Graefelfing, Germany), in a setup with a 180 degree backscattering geometry. A 60× water immersion objective (NA = 1.2) was used in the experiments with cells, and the excitation intensity on the samples was 8 × 10^5^ W cm^−2^. All spectra were obtained with an acquisition time of 1 s, and the spectral resolution was 4–7 cm^−1^ considering the whole recorded spectral range (400–1800 cm^−1^). From 3T3 cells, 489 spectra from 15 cells were obtained for 3 h incubation with NS25, and 232 spectra were obtained from 6 cells for 6 h incubation. From HCT-116 cells, 100 spectra from 8 cells were measured for 3 h incubation with NS25, and 207 spectra were obtained from 7 cells for 6 h incubation. J774 cells spectra analysis was done with 370 spectra from 7 cells exposed to NS25, 252 spectra from 9 cells exposed to NS50, and 226 spectra from 10 cells exposed to NS75, all after an incubation time of 3 h. Processing of the spectra with Matlab R2018a (The MathWorks, Inc., Natick, MA, USA) included frequency calibration, baseline correction, and vector normalization. Band occurrence calculation was performed with Wolfram Mathematica 12 software.

### 2.4. Cryo Soft X-ray Nanotomography

Vitrified cells forming a monolayer with a thickness of up to 10 µm were examined in a transmission X-ray microscope equipped with a cryostage [44]. Microscopy measurements were carried out at beamline U41-PGM1-XM [45] at the electron storage ring BESSY II (Helmholtz-Zentrum Berlin für Materialien und Energie, Berlin, Germany), using a photon energy of 510 eV. The samples were kept at a temperature of −170 °C. Tilt series of the cell samples were acquired at different angle ranges with a 1° increment and a pixel size of 9.8 nm (by using a 25 nm zone plate objective). Depending on the thickness of each sample, the exposure time for each tilt angle was adjusted between 2 and 10 s. Tomograms were obtained by alignment of the corrected tilt series and reconstructed using the Etomo software (IMOD© 4.9.0, CO, USA), either by back-projection or simultaneous iterative reconstruction technique (SIRT). Intracellular gold nanostars were used as fiducial markers for the alignment of the tilt series images.

## 3. Results and Discussion

### 3.1. Optical and Morphological Properties of Gold Nanostars

Gold nanostars for biological applications were synthesized using HEPES, a common buffer in biochemical studies, to ensure biocompatibility [46,47]. HEPES acts as a reducing [48] and a growth director agent [49,50], and depending on the HEPES concentration used, the optical and morphological properties of the nanostars can be tuned [23,49,51]. For brevity, the nanostars obtained with different HEPES concentration are termed here NS25 when synthesized with 25 mM HEPES, NS50 when obtained with 50 mM HEPES, and NS75 when prepared with 75 mM HEPES. Transmission electron microscopic (TEM) images of the nanostars show an increasing spike length when increasing the reducing agent concentration (Figure S1), as well as the formation of spike branches for the highest concentration used (NS75, Figure S1). The absorbance spectra of the nanostars show the presence of two bands related to the surface plasmon resonances of the nanostructures (Figure S2). The band at lower wavelengths at 537 nm can be associated with a plasmon mode of the inner core [1], which is similar for all spectra measured. At longer wavelengths, a band related to the star tips´ resonance [1] can be observed, that shows a red-shift with increasing HEPES concentration. This observed shift is in line with the variations in morphology of the nanostars observed by TEM, as previously reported [23]. As shown previously, given the nanostar synthesis approach, the gold surface remains available for interaction with the surrounding media [23], therefore allowing for the formation of a protein corona and subsequent modification by the processing in cells.

### 3.2. Nanostar–Biomolecule Interactions Differ in Different Cell Lines

Cells from the three different cell lines, 3T3, HCT-116, and J774, were incubated with NS25 for three hours. All cell lines have been studied extensively by SERS before using spherical gold nanoparticles of different size [31,33,35,52]. The mouse fibroblast 3T3 and human epithelial carcinoma HCT-116 cell lines were recently shown to internalize by endocytosis HEPES-synthesized nanostars [23] that are used here from the surrounding culture media, and the interaction of the nanostructures with the biomolecules of the surrounding intracellular environment could be assessed with SERS spectral analysis.

**Figure 1:**
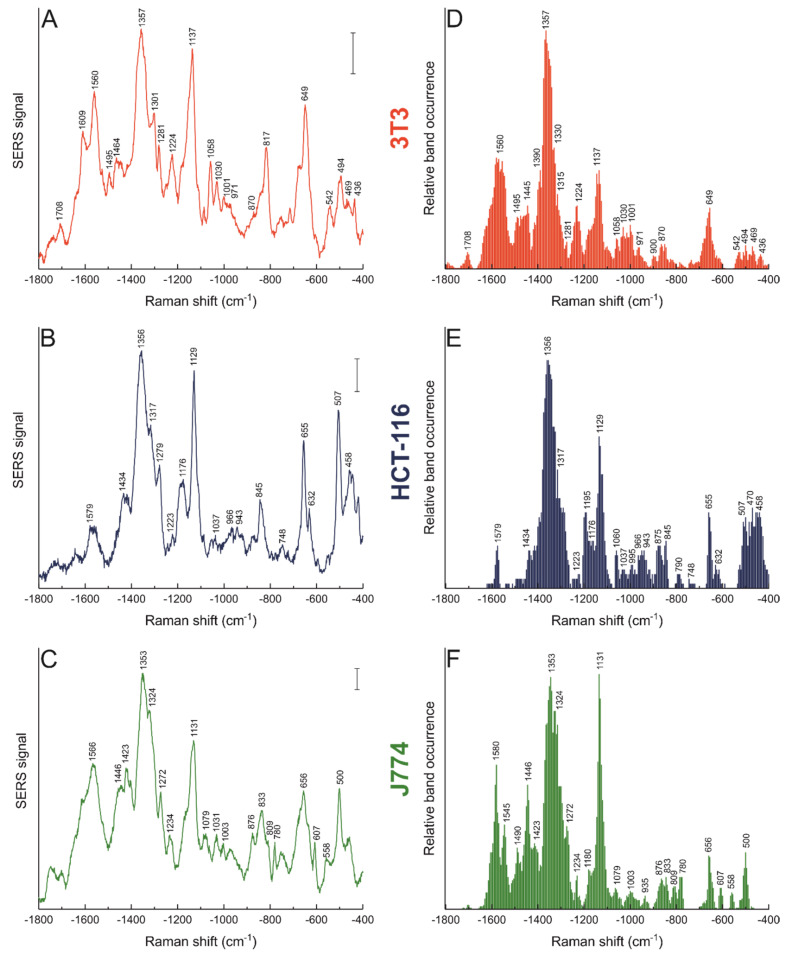
SERS average spectra (left) and relative band occurrence (right) for 3T3 cells (**A**,**D**), HCT-116 cells (**B**,**E**), and J774 cells (**C**,**F**) incubated with gold nanostars synthesized with 25 mM HEPES (NS25) for 3 h. Tentative band assignments provided in Table 1. Scale bars (**A**–**C**): 5 cps.

SERS spectra of several different cells from each cell line were measured. The average spectra for 3T3, HCT-116, and J774 cells are shown in Figure 1A–C. Since the signal intensity or the presence of any band in an average spectrum does not necessarily reflect how frequently a band is found throughout the data set, the band occurrence of the spectra of each cell line was also calculated (Figure 1D–F). With this information it is possible to evaluate the most frequent interactions of molecular functional groups with the nanostar surface, and to determine together with the average spectra the predominant features of the nanostructure–biomolecule interaction in the samples, in this case in the intracellular environment. Band assignments are listed in Table 1. As can be seen in Figure 1, both the average spectrum and the relative band occurrence for each cell line show distinct features that account for the different biomolecules that interact with the nanostars after the incubation period of three hours, corresponding to the age of the oldest endosomes containing nanostars that are present. Nevertheless, there are bands that are common in the spectra of the three cell lines, which can be associated with particular functional groups, mostly related to aromatic amino acid residues. The tryptophan band at 1353 cm^−1^ is the most frequent across all three different types of cell, and bands that can be assigned to tyrosine at 1030 cm^−1^ and to phenylalanine, at ~1000 cm^−1^ and 1030 cm^−1^ are also observed, albeit with a much lower occurrence (compare Figure 1D–F). This shows that the hydrophobic interactions of the nanostars with the proteins in the surrounding environment are a common feature, and do not depend on the type of cells that uptake the nanostructures.

For a discussion of the protein–nanostructure interaction, it is of particular interest to discuss the presence of bands that can be associated with an intact secondary structural element in the proteins interacting with gold nanoparticles [58], as well as those indicating interactions with protein fragments in the vicinity of the nanostructures [32]. The stretching modes of S-S and C-S bonds at ~500 and ~650 cm^−1^, respectively, are common to all cell lines, but they occur with a different frequency in comparison, being much more frequent for HCT-116 cells (compare Figure 1D–F). When comparing J774 and HCT-116 cells (compare Figure 1E,F), protein backbone signals are much more frequent in the J774 macrophages, and side chain bands are more prominent for the HCT-116 epithelial carcinoma cells. Aromatic amino acid side chains show additional features in HCT-116 cells as well, with frequent bands appearing in the 1175–1200 cm^−1^ region (compare Figure 1E,F and Table 1). In the case of J774 (Figure 1F), the amide II and amide III regions present a high frequency of bands in comparison to HCT-116 (Figure 1E, Table 1), including also bands that indicate interactions with CH_2_/CH_3_ groups, e.g., 1272, 1324, and 1446 cm^−1^, that show higher occurrence in J774 as well. Moreover, the band associated with C-C and C-N protein backbone vibrations at ~1130 cm^−1^ is much more prominent in the spectra of the macrophages (compare Figure 1E,F).

Overall, the band occurrence in J774 cells for the nanostars is similar to that observed for incubation of these macrophage cells with spherical gold nanoparticles for a slightly shorter time of 1.5 h [32]. Based on a previously reported systematic study employing a protein model to assess fragmentation of the protein corona in the endosomes of J774 macrophages [32], the high frequency of occurrence of amide-related signals in the macrophages here (Figure 1F) suggests that in the endosomes of the macrophages after this incubation time, the proteins interacting with the nanostars are less fragmented. In contrast, the high occurrence of aromatic amino acid bands in HCT-116 points towards more advanced protein denaturation/fragmentation there. In addition, when comparing the spectra of the macrophages with those collected from the 3T3 cells containing NS25 (Figure 1D), the C-C and C-N protein backbone vibrations at ~1130 cm^−1^ occur less frequently in the fibroblast cells (compare Figure 1D,F). Similar to the data from HCT-116, many aromatic amino acid bands, associated with phenylalanine (1003 cm^−1^) and tryptophan (1224 cm^−1^), and carboxylate vibrations of acidic amino acids (1708 cm^−1^) appear much more frequently in the fibroblast spectra (compare Figure 1D,F). Nevertheless, the bands associated with amide II vibrations are observed with similar frequency in the fibroblast and the macrophage cell lines. These data suggest that, during the 3 h incubation time, the degree of fragmentation of the proteins that the nanostars interact with in 3T3 cells represents an ‘intermediate’ stage between what can be observed for J774 and HCT-116, where the interaction with the protein backbone is less frequent and bands associated with hydrophobic side chains become more prominent.

The data indicate that in all cell lines the nanostars mainly display interactions with proteins, indicating the formation of a protein corona [31,32], and that this protein corona displays a different degree of fragmentation in each cell line. These observations are most probably due to different time frames required for uptake, maturation of endosomes, and fusing with lysosomes in each cell line that would lead to such differences in the fragmentation or denaturation of the proteins interacting with the nanostars. It may also point towards a very different composition of the adsorbed proteins, as well as to different endosomal properties that vary, specifically due to other uptake mechanisms that are in place in macrophage cells and that include fusion of the endosomes with other types of vesicles, such as phagosomes [59].

Interestingly, many bands in the spectra of the macrophages indicate a more pronounced interaction of the nanostars with non-polar side chains and lipids in the macrophages when compared to 3T3 cells. Although lipid signals are not absent from the spectra in the 3T3 cells, cf. the bands at 430 and 540 cm^−1^ that can be related to vibrations of cholesterol (Figure 1D), bands that can be assigned to different CH deformation modes at 1270, 1423, and 1446 cm^−1^ (Figure 1E, Table 1) [54], are found more frequently in the environment of the nanostars in the macrophages, suggesting their more pronounced interaction with non-polar side chains and lipids. Moreover, bands of skeletal C-C and C-N stretching vibrations of lipids, e.g., at 1079 cm^−1^ [54], are observed in macrophages that do not occur in the other cell lines (compare Figure 1D–F). Bands assigned to O-P-O stretching vibrations of nucleic acids at 780 and 809 cm^−1^ are observed in J774 cells (Figure 1F), while a band at 817 cm^−1^ that can be associated with the same type of vibration is found in the average spectra of 3T3 cells (Figure 1A) but at a much lower frequency of occurrence (Figure 1D). As nucleic acids generally do not reside in such transport vesicles in the endolysosomal system, these results suggest that the nanostars are located in digestive organelles or organelles targeted for exocytosis, which is in agreement with the high amount of histones found in the protein corona of spherical gold nanoparticles in J774 cells [32,60]. The differences observed for the lipid bands, together with the observed variation of protein integrity, indicate that in their transit in the endolysosomal pathway, the nanostars interact with several different types of components of the intracellular environment, and that depending on the cell line their uptake and processing varies.

### 3.3. Nanostar Processing and Distribution in Cellular Compartments

The spectral signatures of cells incubated with the gold nanostars indicate that the chemical identity of the interacting functional groups at the gold surface is different for each cell line. Since these differences are observed on a molecular level that concerns mainly the protein corona of the nanoparticles, it is interesting to evaluate if they reflect also differences on an ultrastructural level, where the nanostar uptake and distribution—mediated by the protein corona—inside the cells is affected. To this end, NS25-incubated cells from the three cell lines were plunge-frozen for analysis with cryo soft X-ray nanotomography (SXT). SXT allows for the observation of the intact ultrastructure of cells in a near-native state, and the nanoparticle distribution is easily determined, due to the high absorption coefficient of the metal compared to the organic components [61] of the cell.

**Figure 2:**
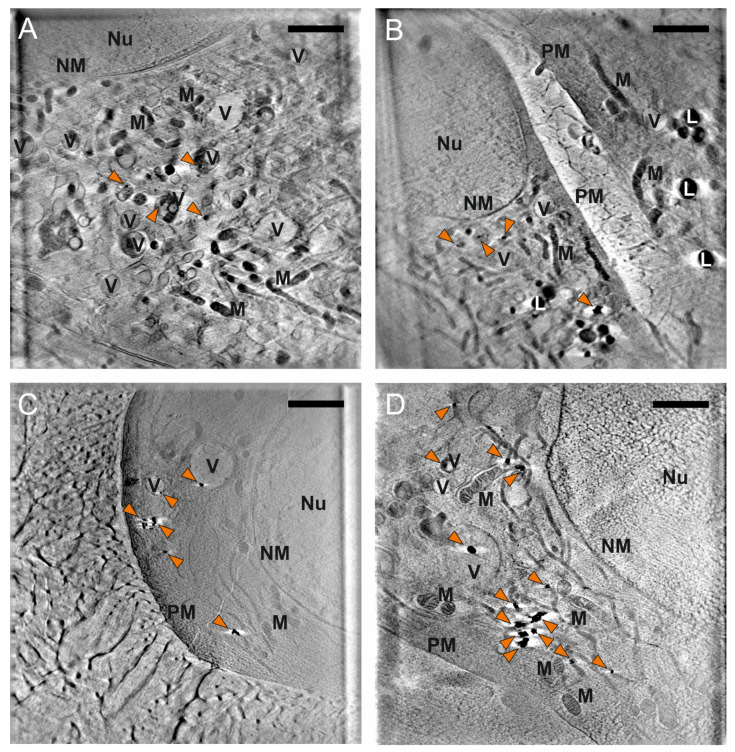
Slices of X-ray tomographic reconstruction of 3T3 (**A**,**B**) and J774 (**C**,**D**) cells incubated for 3 h with gold nanostars synthesized with 25 mM HEPES (NS25). The orange arrows indicate single particles or aggregates of nanostars. More examples are provided in Figure S3. Abbreviations: Nu, nucleus; NM, nuclear membrane; V, vesicles; M, mitochondria; L, lipid droplets; PM, plasma membrane. Scale bars: 2 µm.

Slices of the X-ray tomographic reconstructions for the 3T3 and J774 cells are shown in Figure 2 (see Figure S3 for additional examples). For 3T3 cells, the nanostars are mainly found inside vesicles, as single particles or in very small aggregates that comprise not more than five nanostars (Figure 2A,B, Figure S3A,B). This is very different from such data obtained after long incubation times of 3T3 cells with nanostars for 24 h that we reported in previous work [23], that give rise to much larger aggregates. The observation of many individual particles after 3 h incubation indicates that the uptake in this cell line occurs for individual particles rather than aggregates formed in the extracellular space. There is no particular preference in terms of localization of the nanostars with respect to the cellular ultrastructure, and individual particles or aggregates enclosed in vesicles can be found close to the membrane, close to the nucleus, or anywhere in the cytoplasm. This is different from what was observed for the same cell line incubated with spherical nanoparticles for 3 h, where individual particles and small aggregates were found mainly in the perinuclear region, and larger aggregates observed near the plasma membrane [33]. Since the nanoparticle processing by the cells generates their intraendosomal aggregation [33], the differences in distribution and aggregation degree must be due to a different processing of the particles in the endolysosomal pathway, and/or to a different rate of uptake. In both cases, the main distinction is the shape of the particles, making it clear that differences in particle geometry have an important effect in the processing pathways by the cells.

In the case of J774 cells incubated with nanostars NS25 for 3 h (Figure 2C,D, Figure S3C,D), no individual particles are found, and the nanostars are observed in aggregates ranging from 80 to 550 nm in their longer direction, with aggregates not necessarily being isotropic. The distribution of the aggregates is homogeneous throughout the volume of the cytoplasm, with both small and large aggregates found near the nucleus and the plasma membrane. A similar result was observed when incubating J774 cells for 3 h with spherical gold nanoparticles [33]. Although it is not always clear from the X-ray microscopic images or tomographic reconstruction slices whether all aggregates are in vesicles, the data here show that many small aggregates comprising not more than 5–6 nanostars can be found in close contact with their containing vesicles’ membrane, which is in agreement with the presence of lipid-associated bands in the spectra from these cells (Figure 1C,F). In comparison to what was observed for 3T3 cells, the different morphology of the nanoparticles here (comparing to spherical nanoparticles) [33] does not seem to impact their uptake and processing to a great extent, and neither does it affect their intracellular interactions with biomolecules [32].

For HCT-116 cells incubated with nanostars NS25, displayed for completeness in Figure S4, only a few differences can be found for the short (3 h) incubation time used in these experiments here and the long incubation times of 24 h reported previously [23]. The sections of the tomographic reconstruction (Figure S4A,B) show the presence of aggregates between 90 and 450 nm, with prevalence of aggregate lengths of up to 250 nm. The size range for these aggregates is smaller than what was observed for longer incubation times with the same nanostars [23], but their distribution across the cytoplasm is equally homogeneous. These results suggest that the uptake and processing of the nanostars depends on the particular cell line, and the differences between the different types of cells can be seen already after relatively short incubation times, that is, in earlier stages of endosomal maturation. Specifically, the degree of aggregation of the nanostars in the intracellular space differs for the different cell lines, most possibly due to differences in the rate and mechanisms of uptake. This is in agreement with the different SERS spectra obtained and the observed features.

### 3.4. Morphology Dictates Nanostar–Biomolecule Interaction

**Figure 3:**
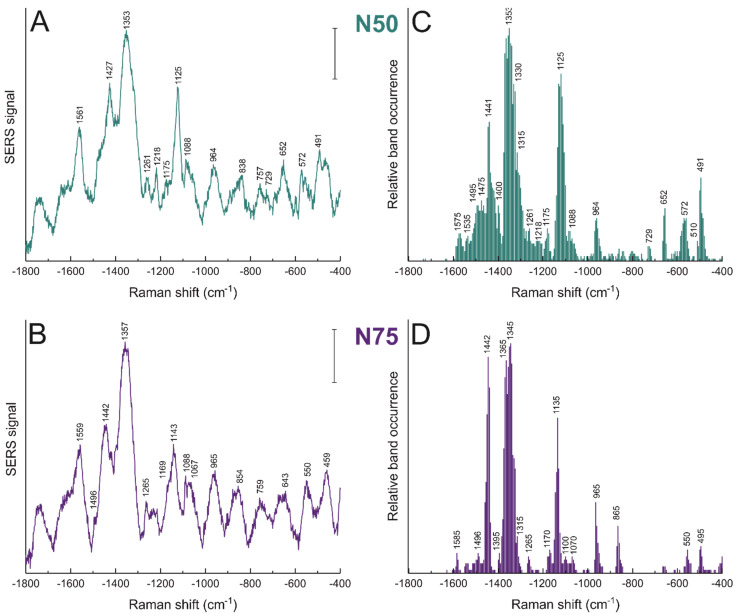
SERS average spectra (left) and relative band occurrence (right) for J774 cells incubated for 3 h with gold nanostars synthesized with 50 mM HEPES (**A**,**C**; NS50) and 75 mM HEPES (**B**,**D**; NS75). Tentative band assignments provided in Table 2. Scale bars (**A**,**B**): 5 cps.

To study potential effects of morphology on the processing of the gold nanostars by the macrophage cells, J774 cells were incubated with nanostars of different spike length, NS50 and NS75, for 3 h in their culture medium. The SERS average spectra and the relative band occurrence for the dataset obtained are shown in Figure 3 and the tentative band assignments in Table 2, and the data are also compared to those discussed above in Figure 1C,F that were obtained for NS25. There is a clear decrease in the frequency of occurrence of bands in the amide II and amide III regions with increasing spike length of the nanostars (Figure 1F, Figure 3C,D). Moreover, the signals assigned to aromatic amino acid side chains, including those of phenylalanine at 1003 cm^−1^ and of tyrosine at 840 cm^−1^, become less frequent for NS50 (Figure 3C) and NS75 (Figure 3D) when compared to NS25 (Figure 1F). In cells incubated with NS75, vibrational bands related to disulfide bonds around 500 cm^−1^ and to C-S bonds ~650 cm^−1^ also occur less frequently compared to the two other types of nanostars with shorter spike length (compare Figure 3D with Figure 3C and Figure 1F). The decrease in occurrence of these protein-associated bands with increasing spike length suggests that the interactions with proteins by these nanostars must be less frequent. Although in principle, a decrease in protein signals could also be related to inhomogeneous enhancement, where the proteins preferably interact with those regions of the nanostars that do not present high enhancement due to their anisotropy [62]. The field distribution is quite homogeneous when considering interacting particles, especially for nanostars with longer spikes [12,23]. As revealed by the SXT data discussed above for the gold nanostars with the shorter tips (Figure 2C,D), and suggested by the well-known aggregation also of other types of nanoparticles in the endolysosomal compartment of this cell line [31,33], the absence of bands associated with certain biomolecular species is more likely to occur due to a lack of interaction than due to a diminished enhancement of protein signals. In support of our interpretation of the in vivo data here, it has been demonstrated that changes within a defined shape in the overall morphology, that is, different spike lengths in nanostars, can give rise to protein reorientation in vitro [63].

In addition to a different interaction with proteins, bands associated with lipid structures also have a higher frequency of occurrence with increasing spike length of the nanostars. Bands related to characteristic vibrations of lipids, such as the C-N stretching at 865 cm^−1^, the C-C stretching at 965 cm^−1^, or deformations in the lipid chains around 1440 and 1365 cm^−1^ (Table 2) occur more frequently in NS75 compared to NS25 (compare Figure 3D with Figure 1F). In addition, other examples of an increased contribution of lipid signals to the SERS spectra, such as the band at 1125 cm^−1^, assigned to lipid C-C vibrations in NS50 (Figure 3C), point towards a more intimate interaction of lipids, possibly from the membrane of the endolysosomes, with the nanostars when the length of the nanostar spikes increases. The results reported here are in agreement with those found for fibroblast cells with longer incubation periods, where the nature of the interactions between biomolecules in the endolysosomes and the nanostars changed with spike length in a similar fashion, with more hydrophobic interactions and lipids in the proximity of the nanostar surface [23]. The differences in the interactions observed between proteins and lipids in the macrophages here, even when the geometry variation is only small, show that the foundations of these interactions are independent of the cell line and are related to the shape of the nanostructure.

### 3.5. Nanostar–Biomolecule Interaction Variability with Time

Different from experiments where gold nanoparticles are delivered into cells in a defined incubation pulse and later the development of their surface species in the course of their processing by the cells can be monitored [35,52,55], the incubation process of the nanostars with the cells that allows for the endocytic uptake of the gold nanostars here consists of a continuous exposure of the cells to a nanostar suspension in the culture medium. At the time of the SERS experiment, they are in any stage of their processing in the endolysosomal pathway from the earliest time point after uptake to the maximum processing time that corresponds to the incubation time. Therefore, it is not possible to determine the exact step in the endolysosomal pathway in which nanostars are based on the obtained spectra. Nevertheless, the spectral and band occurrence profiles are not identical for different incubation times with nanostars of the same morphology. This indicates that, in addition to the ‘oldest’ endolysosomes aging more with longer continuous incubation, the distribution of nanostars in endolysosomes of different stages also varies.

When 3T3 cells are incubated with NS25 for 24 h as reported previously [23], the interactions with skeletal protein bonds and with hydrophobic amino acid side chains become much more frequent than observed in the 3 h incubation time here (Figure 1D). Interestingly, in an intermediate incubation time of 6 h (Figure S5, Table S1), some of the above-mentioned bands, including the disulfide stretching and vibrations of the hydrophobic side chains of tryptophan and tyrosine (Table S1), already begin to appear more frequently in the SERS spectra (Figure S5B), and overall show features similar to the band occurrences observed for a 24 h incubation [23]. This similarity suggests that the SERS signals after 6 h of incubation present features that comprise a more complete depiction of all different endolysosomal processing stages, while incubation times as short as 3 h might show preferentially early stages of the nanostar processing, lacking the late endosomal and lysosomal stage. This is further supported by the X-ray nanotomography results (Figure 2A,B, Figure S3A,B), where a 3 h incubation time of 3T3 cells with nanostars yields endosomes containing individual particles or small aggregates, indicating an early stage of particle processing in the endolysosomal pathway.

In contrast, the development of the band occurrences in the spectra of the HCT-116 cells over time is very unalike that in the 3T3 fibroblast cells, when the same incubation times are compared (Figure S6). After 6 h (Figure S6, Table S1), the interactions with disulfide and C-S bonds are less frequent than after 3 h incubation (compare Figure S6B with Figure 1E), but the bands in the amide II region and of methyl deformations become more prominent. In addition, and also opposed to the observation in 3T3 discussed above, bands assigned to vibrations of aromatic amino acid residues are found less frequently (compare Figure S6B with Figure 1E), e.g., at 845 cm^−1^, assigned to tyrosine, at 1000 cm^−1^ of phenylalanine, and at 1195 cm^−1^. This indicates a change in the nature of the interactions of proteins with the nanostars in the endolysosomes of HCT-116, with protein backbone interactions becoming more prominent as opposed to hydrophobic interactions of the side chains. However, as reported previously for this cell line as well, after 24 h of incubation amide II bands, as well as bands in the amide III region, are much less pronounced [23], and many of the protein-related vibrations that decrease in frequency for 6 h incubation (Figure S6B) are more frequently found again after longer incubation times in the SERS spectra of the HCT-116 cells [23]. Additionally, interactions with lipids become more important with longer incubation times [23]. The differences in the surface composition of the nanostars after the relatively short incubation time, but moreover those found for incubation times of 6 h, where endolysosomes of later stages must be present, provide an explanation for the great differences in the distribution and morphology of the nanostar aggregates that are found by nanotomography after long incubation times, as reported previously [23].

## 4. Conclusions

The data obtained with fibroblast cells, epithelial cells, and macrophages clearly indicate that the processing of gold nanostars varies depending on a specific cell line. Therefore, they support a first, recent report, where we observed differences in the corona of the nanostars with respect to hydrophobic interactions in the cell lines 3T3 and HCT-116 after long incubation times of 24 h [23]. The results here convey that significant differences in the interaction of proteins in the endolysosomal environment of these two non-phagocytic cell lines occur already at a relatively early stage after an incubation time of 3 h, before differences in aggregate morphology and distribution can be observed by nanotomography that manifest only in endolysosomes of later maturation stages [23]. The biomolecular corona and the interaction of the nanostars in the endosomal system of macrophage cells of the cell line J774 were found to be entirely different from the two non-phagocytic cell lines, in agreement with phagocytic uptake mechanisms in place that influence the processing of endocytic vesicles as well. Similarities of the SERS spectra with those obtained with spherical nanoparticles in the same macrophage cell line [32] were identified, suggesting that the structure and composition of the biomolecular corona of the nanostars in these cells resembles theirs, and that the proteins in the corona must be processed in a similar fashion. Moreover, the SERS spectra obtained from the macrophage cells are indicative of an increased interaction of the nanostars with lipid components of the endolysosomes, and nucleic acids that account for digestive or exocytosis-targeted vesicles.

Incubation of the macrophage cells with nanostars of different spike length yielded SERS fingerprints that revealed varied interactions with the surrounding intracellular biomolecules. Spectra obtained after incubation with nanostars of longer spikes were indicative of more hydrophobic interactions and lipids in the proximity of the gold surface at the early stages of uptake and processing that were studied here. The differences in the interaction that were found for the three types of nanostars in the macrophages resemble those in fibroblast cells reported previously [23], underlining that they are caused by the different shape of the nanostructure that leads to the formation of a biomolecular corona of different composition, and ultimately to different interactions with/in the endosomal ultrastructure. The results presented here extend our knowledge on the endolysosomal processing of gold nanostars by animal cells, with implications for further developments based on such structures in bioanalysis, biotechnology, and theranostics.

## Figures and Tables

**Table 1:** Raman shifts and their tentative assignments in the spectra displayed in Figure 1. Band assignments based on ref [23,31,32,52,53,54,55,56,57]. Abbreviations: Trp, tryptophan; Tyr, tyrosine; Phe, phenylalanine; Cys, cysteine; Pro, proline; Val, valine; Asp, aspartic acid; Glu, glutamic acid; T, thymine; C, cytosine; U, uracil; A, adenine; G, guanine; str, stretching; def, deformation; twist, twisting; br, breathing; bend, bending; wag, wagging; rock, rocking; sciss, scissoring.

Raman Shift (cm^−1^)			Tentative Band Assignment
*3T3*	*HCT-116*	*J774*	
436			Cholesterol
	458	457	Protein S-S str, C-S str, Trp ring def
469	470		C-S str
494	507	500	Protein S-S str
542			S-S str, Cholesterol
		558	S-S str
		607	COO^-^ def, ring def, C-H def
	632		C-S str
649	655	656	Cys C-S str, Tyr C-C twist, Phe
752	748		Trp br; Pro; C-S, C-C str; T
	790	780	O-P-O str; C, U, T ring br
		809	Pro, Tyr, C-C str, lipid O–P–O str
817			O-P-O str; COO^-^ def
	845	833	Tyr ring br, Phe, Cα-N and C-C str, O-P-O str
870	875	876	Pro, Val C-C str; C-N str
900			Trp, C-C, C-N str
		935	Pro, C-C str
	943		C-C str
971	966		Lipids C-C str, Pro, Val
1001	995	1003	Phe ring br
1030	1037	1031	Phe, C-C str, Tyr ring def
1058	1060		Protein C-C and C-N str
		1079	Lipid O-P-O str, C-C str; C-N str
1137	1129	1131	Protein backbone C-C str; C-N str
	1176	1180	Tyr C-H bend; Phe
	1195		Trp, Tyr, Phe; Aromatic C-O and C-N
1224	1223		Amide III; Trp ring
		1234	Amide III, CH_2_ wag, O-P-O str
1281	1279	1272	Amide III; CH/CH_2_/CH_3_ def
1301			Amide III, A and C, CH/CH_2_/CH_3_ def
1315	1317		Amide III, G, lipids CH_2_/CH_3_ def
		1324	Amide III; G; protein CH_2_/CH_3_ twist
1330			Amide III; CH_2_/CH_3_ def
1357	1356	1353	Protein CH/CH_2_/CH_3_ def; Trp
1390			CH rock
		1423	A, G, CH_3_CH_2_ twist
	1434		CH_2_ def
1445		1446	CH_2_/CH_3_ def
1464			CH_2_ def
1495		1490	Amide II, NH_3_^+^
		1545	Amide II, lipid CH_2_ sciss
1560		1566	Amide II; Trp, Tyr, COO^–^ str
1575	1579	1580	Amide II, C-C str,COO^–^ str
1609			Trp, Tyr, Phe
1708			Asp, Glu C=O str

**Table 2:** Raman shifts and their tentative assignments in the spectra displayed in Figure 3. Band assignments based on ref [23,31,32,52,53,54,55,56,57]. Abbreviations: Trp, tryptophan; Tyr, tyrosine; Phe, phenylalanine; Cys, cysteine; Pro, proline; Val, valine; T, thymine; C, cytosine; A, adenine; G, guanine; str, stretching; def, deformation; twist, twisting; br, breathing; bend, bending; rock, rocking.

Raman Shift (cm^−1^)		Tentative Band Assignment
*NS 50*	*NS 75*	
	459	Protein S-S str, C-S str, Trp ring def
491	495	Protein S-S str
	550	S-S str
572		Trp
652		Cys C-S str, Tyr C-C twist, Phe
729		Lipid C-N str; C-S str
757		Trp br; C-N str
838		Tyr ring br, Phe, Cα-N and C-C str, O-P-O str
	854	Tyr ring br; Phe, Cα-N and C-C str
	865	Lipid C-N str
964	965	Lipids C-C str, Pro, Val
	1067	C-C and C-N str; DNA/RNA O-P-O str; Pro
1088		Lipid C-C str
	1100	Phe; C-N str
1125		Lipid C-C str
	1135	Protein backbone C-C str; C-N str
	1143	Lipid C-C str; C-N str
	1169	Tyr and lipids C-H bend
1175		Tyr C-H bend; Phe
1218		Amide III; T, A; O-P-O str; C-N str
1261	1265	Amide III
1315	1315	Amide III, G, lipids CH_2_/CH_3_ def
1330		Amide III; CH_2_/CH_3_ def
1353	1357	Protein CH/CH_2_/CH_3_ def; Trp
	1365	Lipids CH_2_ def
1400	1395	CH rock; C=O str
1427		A, G, CH_3_CH_2_ twist
1441	1442	CH_2_/CH_3_ def
1475		Lipid CH_2_/CH_3_ def
1495	1496	Amide II, NH_3_^+^
1535		Amide II; N-H def
1561	1559	Amide II; Trp, Tyr, COO^–^ str
1575		Amide II, C-C str,COO^–^ str
	1585	C=C str, COO^–^ str; Phe

## Data Availability

Data are available from the authors upon reasonable request.

## Supplementary Materials

The following are available online at https://www.mdpi.com/article/10.3390/nano11051183/s1, Figure S1: TEM micrographs of gold nanostars, Figure S2: UV-vis-NIR spectra of gold nanostars, Figure S3: Slices of X-ray tomographic reconstruction of 3T3 and J774 cells incubated for 3 h with NS25, Figure S4: Slices of X-ray tomographic reconstruction of HCT-116 cells incubated for 3 h with NS25, Figure S5: SERS average spectra and relative band occurrence for 3T3 cells incubated for 6 h with NS25, Figure S6: SERS average spectra and relative band occurrence for HCT-116 cells incubated for 6 h with NS25, Table S1: Raman shifts and their tentative assignments in the spectra displayed in Figures S5 and S6.
