# Supplementary material for: Probing the Intracellular Bio-Nano Interface in Different Cell Lines with Gold Nanostars
Source: Nanomaterials (Basel). 2021 Apr 30;11(5):1183. doi: 10.3390/nano11051183 (PMC8145934; doi:10.3390/nano11051183)
Supplement: Supplementary file 1 [file nanomaterials-11-01183-s001.zip › nanomaterials-1196446-supplementary.pdf]

nanomaterials

Article

Probing the Intracellular Bio-Nano Interface in Different Cell Lines with Gold Nanostars

Cecilia Spedalieri [1], Gergo Peter Szekeres [1,2], Stephan Werner [3], Peter Guttmann [3] and Janina Kneipp [1,2,*]

[1] Humboldt-Universität zu Berlin, Department of Chemistry, Brook-Taylor-Str. 2,12489 Berlin, Germany; cecilia.spedalieri@hu-berlin.de (C.S.); gpszekeres@fhi-berlin.mpg.de (G.P.S.); janina.kneipp@chemie.hu-berlin.de (J.K.)
[2] Humboldt-Universität zu Berlin, School of Analytical Sciences Adlershof, Albert-Einstein-Str. 5-9, 12489 Berlin, Germany
[3] Helmholtz-Zentrum Berlin für Materialien und Energie GmbH, Department X-ray Microscopy, Albert-Einstein-Str. 15, 12489 Berlin, Germany; stephan.werner@helmholtz-berlin.de (S.W.); peter.guttmann@helmholtz-berlin.de (P.G.)
* Correspondence: janina-kneipp@chemie.hu-berlin.de

Citation: Spedalieri, C.; Szekeres, G. P.; Werner, S., Guttman, P., Kneipp, J. Probing the Intracellular Bio-Nano Interface in Different Cell Lines with Gold Nanostars. *Nanomaterials* **2021,** *11*, 1183. https://doi.org/10.3390/nano11051183

Academic Editor: Kerstin Leopold

Received: 8 April 2021
Accepted: 27 April 2021
Published: 30 April 2021

Publisher's Note: MDPI stays neutral with regard to jurisdictional claims in published maps and institutional affiliations.

Copyright: © 2021 by the authors. Submitted for possible open access publication under the terms and conditions of the Creative Commons Attribution (CC BY) license (https://creativecommons.org/licenses/by/4.0/).

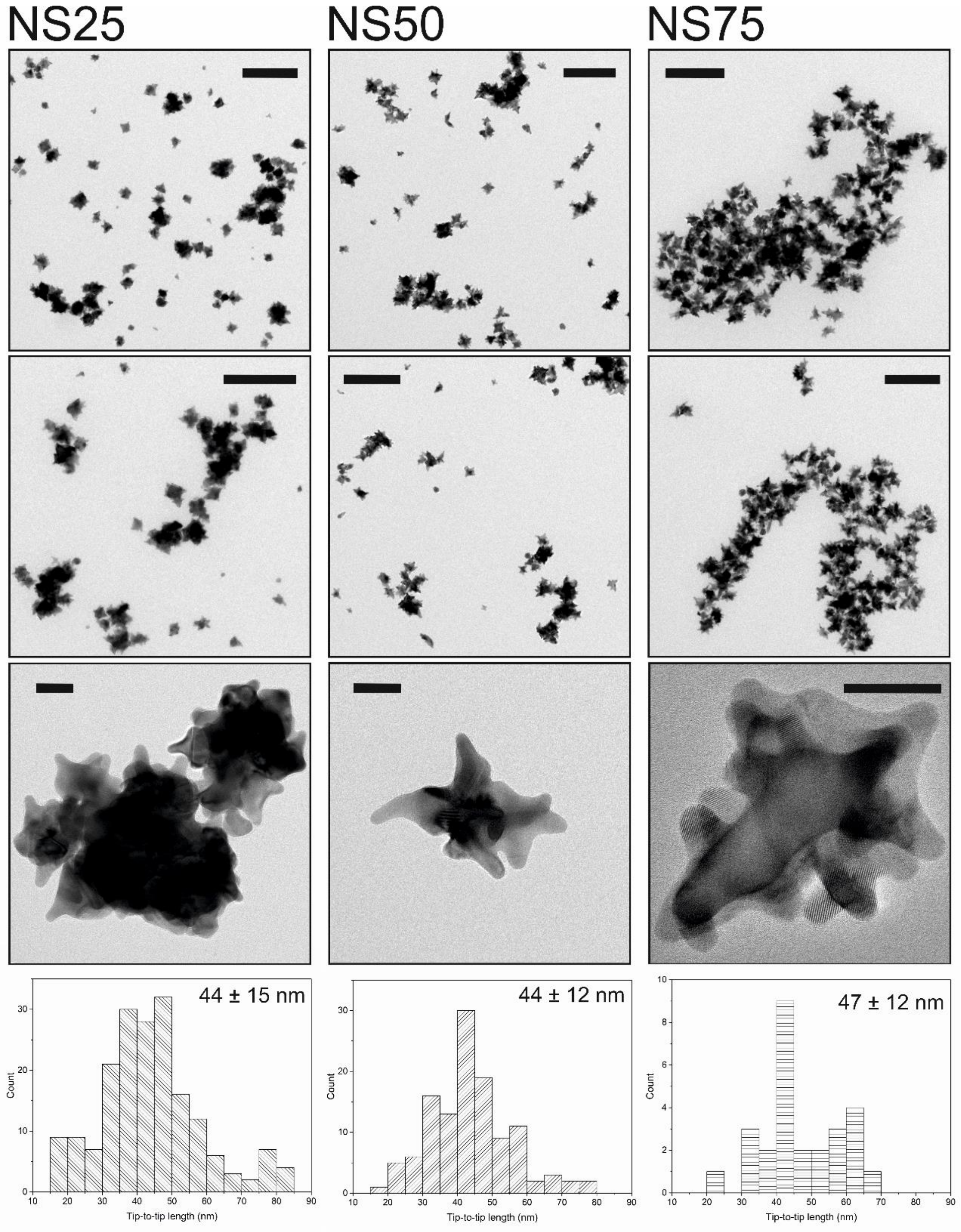

Figure S1. TEM micrographs (first, second and third row) and tip-to-tip length distribution based on TEM data (last row) of gold nanostars synthesized with 25 mM HEPES (NS25, left column), 50 mM HEPES (NS50, middle column) and 75 mM HEPES (NS75, right column). Scale bars first and second row: 200 nm; third row: 20nm. The size distribution average and standard deviation were obtained from 187 particles for NS25, 126 particles for NS50 and 26 particles for NS75.

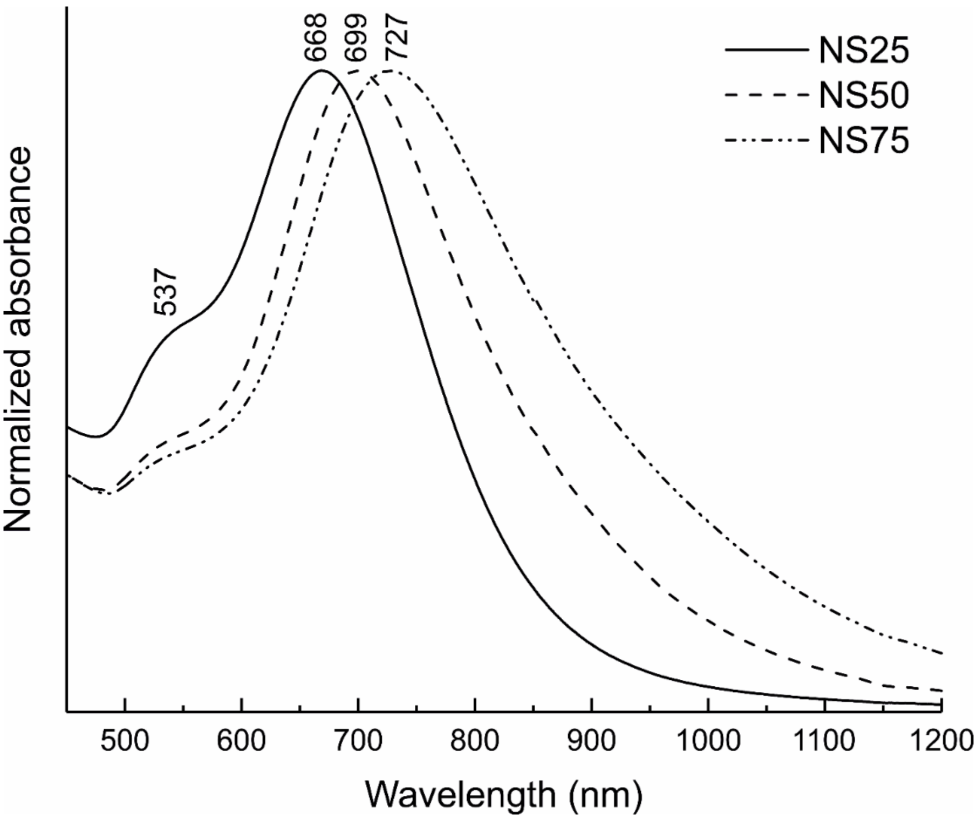

Figure S2. UV-vis-NIR spectra of gold nanostars synthesized with 25 mM HEPES (NS25), 50 mM HEPES (NS50) and 75 mM HEPES (NS75). Plasmon resonance maxima are labeled. The 537 nm band is common to all spectra.

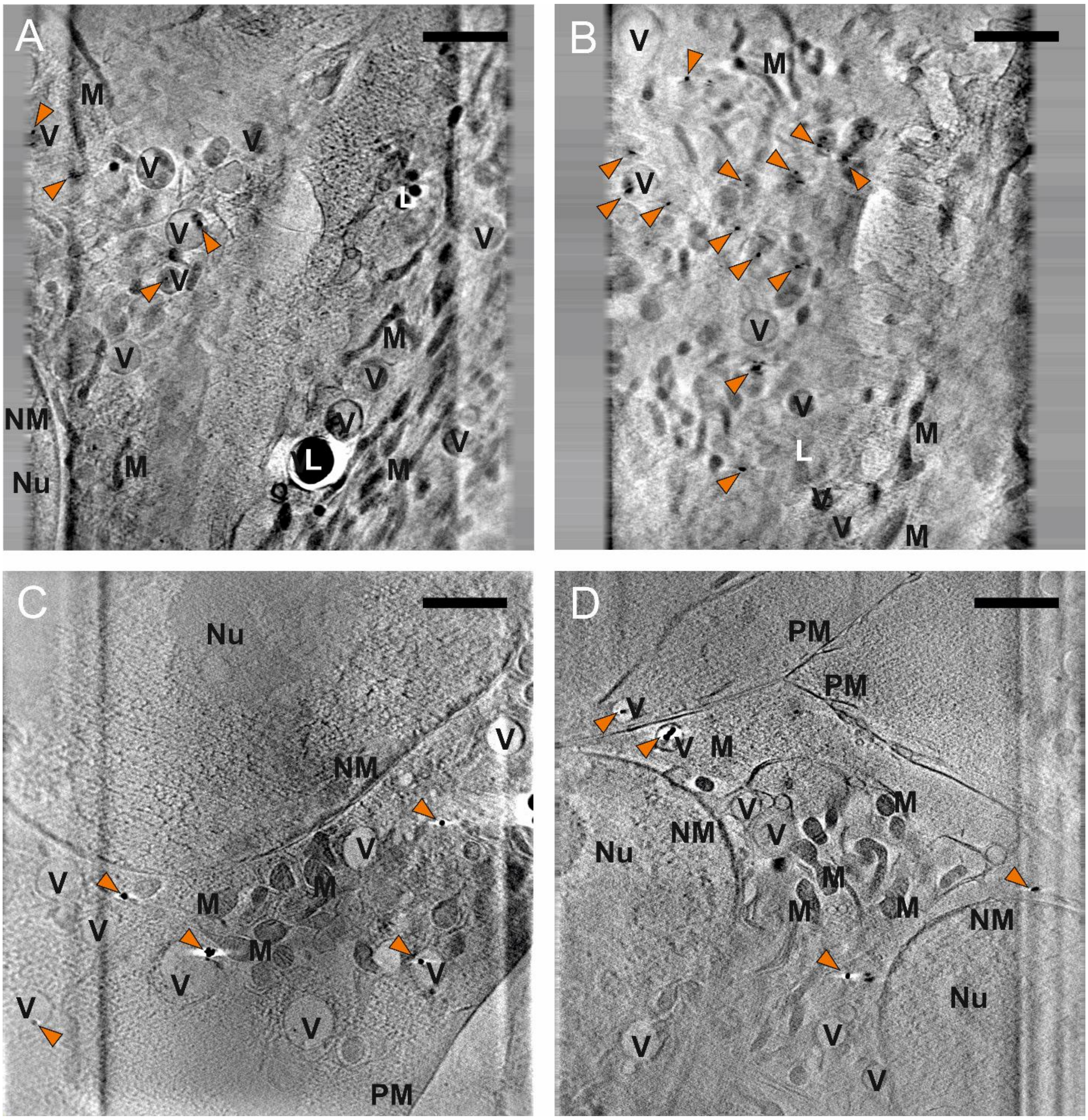

Figure 3. Slices of X-ray tomographic reconstruction (further examples) of 3T3 (**A** and **B**) and J774 (**C** and **D**) cells incubated for 3 h with gold nanostars synthesized with 25 mM HEPES (NS25). The orange arrows indicate single particles or aggregates of nanostars. Abbreviations: Nu nucleus; NM nuclear membrane; PM plasma membrane; V vesicles; M mitochondria; L lipid droplets. Scale bars: 2 μm.

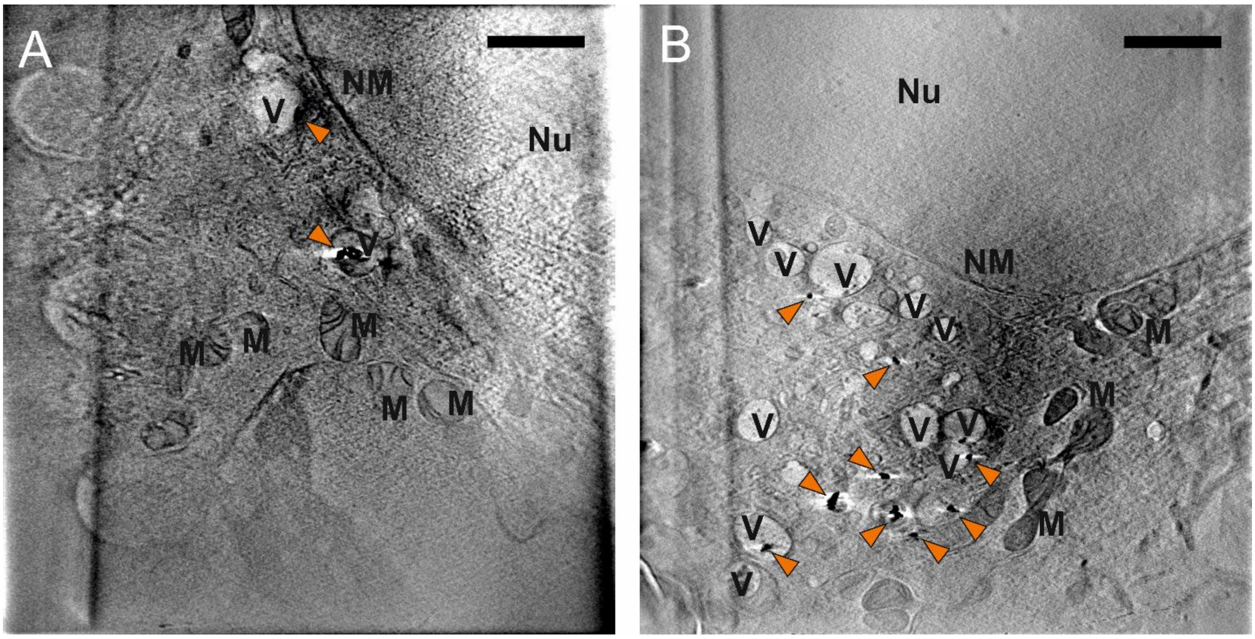

Figure S4. Slices of X-ray tomographic reconstruction of HCT-116 cells (**A** and **B**) incubated for 3 h with gold nanostars synthesized with 25 mM HEPES (NS25). The orange arrows indicate single particles or aggregates of nanostars. Abbreviations: Nu nucleus; NM nuclear membrane; V vesicles; M mitochondria. Scale bars: 2 µm.

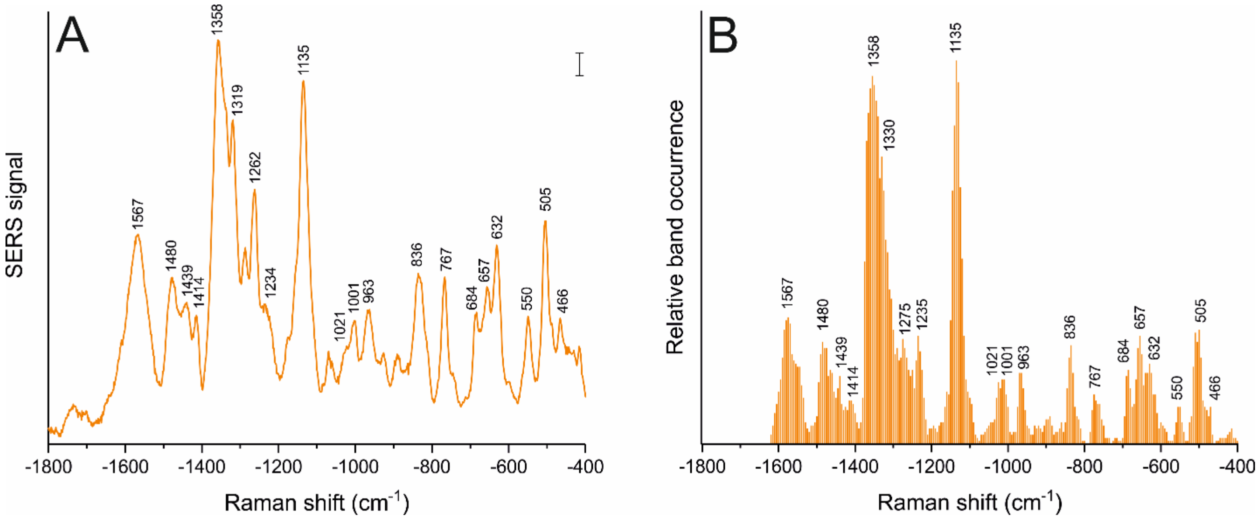

Figure S5. (A) SERS average spectra and **(B)** relative band occurrence for 3T3 cells incubated with gold nanostars synthesized with 25 mM HEPES (NS25) for 6 h. Scale bar in A: 5 cps.

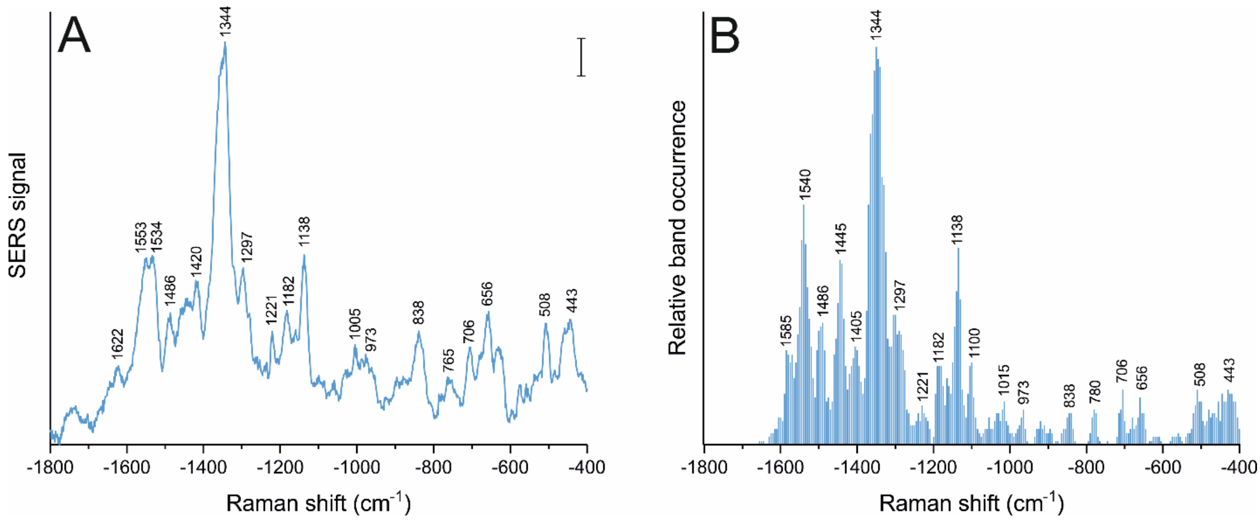

Figure S6. (A) SERS average spectra and **(B)** relative band occurrence for HCT-116 cells incubated with gold nanostars synthesized with 25 mM HEPES (NS25) for 6 h. Scale bar in A: 5 cps.

Table S1. Raman shifts and their tentative assignments in the spectra displayed in Figures S5 and S6. Band assignments and S6. Band assignments based on references[1–9].

**Raman shift (cm^{-1})**		**Tentative band assignment**
3T3	*HCT-116*	
	443	Protein S-S str
466		C-S str
505	508	Protein S-S str
550		S-S str
632		C-S str
657	656	Cys C-S str, Tyr C-C twist, Phe
684		Cys C-S str, Tyr C-C twist
	706	COO$^-$ def, C-S str
767	765	Trp indol br
	780	O-P-O str; C, U, T ring br
836	838	Tyr ring br, Phe, Cα-N and C-C str, O-P-O str
963	973	Lipids C-C str, Pro, Val
1001	1005	Phe ring br

Trp, tryptophan;
Tyr, tyrosine;
Phe, phenylalanine;
Cys, cysteine;
Pro, proline;
Val, valine;
T, thymine;
C, cytosine;
U, uracil;
A, adenine;
G, guanine;
str, stretching;

1021	1015	Ribose C-O str	def, deformation;
	1100	Phe; C-N str	twist, twisting;
1135	1138	Protein backbone C-C str; C-N str	br, breathing;
	1182	Tyr C-H bend; Phe	bend, bending;
	1221	Amide III; Trp ring	wag, wagging;
1234		Amide III, CH_2 wag, O-P-O str	sciss, scissoring
1262		Amide III	
1275		Amide III; CH/CH_2/CH_3 def	
	1297	Amide III, A and C, CH/CH_2/CH_3 def	
1319		Amide III, G, lipids CH_2/CH_3 def	
1330		Amide III; CH_2/CH_3 def	
	1344	CH def	
1358		Protein CH/CH_2/CH_3 def; Trp	
1414	1405	COO^- str	
	1420	A, G, CH_3CH_2 twist	
1439	1445	CH_2/CH_3 def	
1480	1486	Amide II, NH_3^+	
	1540	Amide II, lipid CH_2 sciss	
1567		Amide II; Trp, Tyr, COO^- str	
	1585	C=C str, COO^- str; Phe	
	1622	Trp	

References

1. Talari, A. C. S.; Movasaghi, Z.; Rehman, S.; Rehman, I. u. Raman Spectroscopy of Biological Tissues. *Applied Spectroscopy Reviews*, **2014**, 50, 46-111, doi:10.1080/05704928.2014.923902
2. Szekeres, G. P.; Montes-Bayón, M.; Bettmer, J.; Kneipp, J. Fragmentation of Proteins in the Corona of Gold Nanoparticles As Observed in Live Cell Surface-Enhanced Raman Scattering. *Analytical Chemistry*, **2020**, 92, 8553-8560, doi:10.1021/acs.analchem.0c01404
3. Szekeres, G. P.; Werner, S.; Guttmann, P.; Spedalieri, C.; Drescher, D.; Živanović, V.; Montes-Bayón, M.; Bettmer, J.; Kneipp,J. Relating the composition and interface interactions in the hard corona of gold nanoparticles to the induced response mechanisms in living cells. *Nanoscale*, **2020**, 12, 17450-17461, doi:10.1039/D0NR03581E
4. Živanović, V.; Kochovski, Z.; Arenz, C.; Lu, Y.; Kneipp,J. SERS and Cryo-EM Directly Reveal Different Liposome Structures during Interaction with Gold Nanoparticles. *The Journal of Physical Chemistry Letters*, **2018**, 9, 6767-6772, doi:10.1021/acs.jpclett.8b03191
5. Živanović, V.; Seifert, S.; Drescher, D.; Schrade, P.; Werner, S.; Guttmann, P.; Szekeres, G. P.; Bachmann, S.; Schneider, G.; Arenz, C.; Kneipp, J. Optical Nanosensing of Lipid Accumulation due to Enzyme Inhibition in Live Cells. *ACS Nano*, **2019**, 13, 9363-9375, doi:10.1021/acsnano.9b04001
6. Rygula, A.; Majzner, K.; Marzec, K. M.; Kaczor, A.; Pilarczyk, M.; Baranska, M. Raman spectroscopy of proteins: a review *Journal of Raman Spectroscopy*, **2013**, 44, 1061-1076, doi:10.1002/jrs.4335
7. Czamara, K.; Majzner, K.; Pacia, M. Z.; Kochan, K.; Kaczor, A.; Baranska, M.Raman spectroscopy of lipids: a review. *Journal of Raman Spectroscopy*, **2015**, 46, 4-20, doi:10.1002/jrs.4607
8. Spedalieri,C.; Szekeres, G. P.; Werner, S.; Guttmann, P.; Kneipp, J. Intracellular optical probing with gold nanostars. *Nanoscale*, **2021**, 13, 968-979, doi:10.1039/D0NR07031A
9. Buchner, T.; Drescher, D.; Traub, H.; Schrade, P.; Bachmann, S.; Jakubowski, N.; Kneipp, J. Relating surface-enhanced Raman scattering signals of cells to gold nanoparticle aggregation as determined by LA-ICP-MS micromapping. *Anal Bioanal Chem*, **2014**, 406, 7003-7014, doi:10.1007/s00216-014-8069-0
